# Virtual and Augmented Surgical Skills in Total Hip Arthroplasty

**DOI:** 10.7759/cureus.28895

**Published:** 2022-09-07

**Authors:** Aishwarya P Bhagwat, Dr. Ratnakar Ambade

**Affiliations:** 1 Department of Orthopedics, Jawaharlal Nehru Medical College, Datta Meghe Institute of Medical Sciences, Wardha, IND

**Keywords:** virtual treatment, outcome, surgical training, hip replacement, preoperative templates

## Abstract

A surgical treatment called arthroplasty can be used to rehabilitate the function of joints. Hip arthroplasty is the surgical replacement of a hip joint. It is the procedure in which the joint surface can be replaced, retreated, or readjusted. It is useful when medical treatment cannot overcome long-lasting joint pain. Arthroplasty in patients significantly increases quality of life, activity level, and joint pain. The most commonly performed arthroplasty is of hip and knee joints. During any surgical treatment, complications occur. Some common complications during arthroplasty are hemorrhage, septicemia, mass in the legs and lungs, and loosening of prosthetic parts. This method's proponents point out that quicker recovery periods, decreased pain levels, higher patient satisfaction, and immediate return to function are the highlighted indicators. Many techniques have been created and used for preoperative planning for a hip replacement with varying degrees of effectiveness. It mainly includes digital templating, which has allegedly improved the accuracy of prosthetic implant size prediction and increased the efficacy of total hip arthroplasty (THA). Surgical training for hip replacement includes teaching, practice, treatment, and techniques performed during arthroplasty. Preoperative education also plays a vital role in surgical training as it is a major surgical procedure that is physically and mentally stressful for the patient. The resources in surgical training of hip arthroplasty contribute to rapid progress in surgical training worldwide with increasing occurrence of THA. This article's focus is to draw outcomes from the surgical treatment of hip arthroplasty by comparing and concluding about virtual and surgical treatment.

## Introduction and background

The most prevalent ailment in adults is hip fracture, and arthroplasty is a technique in which the hip joint is replaced to restore proper joint function and treat pathological conditions. This technique has helped a lot of people who had significant hip injuries. Another treatment in hip arthroplasty is called hemiarthroplasty (HA). Studies have demonstrated that total hip arthroplasty (THA) produces superior outcomes than HA [1]. The anterior, lateral anterolateral, and posterior approaches are the most common arthroplasty procedures. One surgical training that includes the surgical technique for hip replacement is THA. THA is the finest surgical treatment for those with advanced hip osteoarthritis THA. The academic and technical abilities necessary for THA are taught to young orthopedic trainees in hospitals and surgical specialty facilities. Due to their lack of surgical expertise, trainees should be overseen by more seasoned and knowledgeable surgeons. Globally, over a million primary THA surgeries are performed each year. However, for adolescent, active patients, THA lifespan is a serious problem. THA has excellent long-term results, according to published literature [2]. Surgical skills are essential in the procedure of hip arthroplasty. THA is a helpful surgical procedure for individuals with advanced hip osteoarthritis. Many authors claimed that THA produced fantastic long-term results [2]. Several governmental efforts are also aimed at enhancing quality by providing incentives for surgeons to follow evidence-based treatment methods. This practice sought to estimate the individual effects of a surgeon, facility, standard of care procedure volume, and resource use in lower extremity THA [3].

## Review

In hip arthroplasty, modern surgical training has been developed, which will be helpful for the progression of the procedure. Patients whose hip joint has been degenerated by traumatic condition or disease may be able to experience pain relief and be back to regular work following a total hip replacement. In this kind of surgery, artificial implants are used to replace the femur ball and the hip socket that is damaged. Recently, surgical methods for hip replacement, particularly anterior (through the front of the hip) versus posterior (through the rear of the hip) treatments, have attracted new interest in the press and the medical community. The three primary forms of hip replacement are partial hip replacement, hip remerges, and THA. An operation known as arthroplasty can be performed to restore function to a joint. A joint may be rehabilitated by resurfacing the bones.

Surgical training

The surgical procedure involving implanting a complete hip prosthesis is a THA. It is performed in joint destruction due to degeneration or trauma. In our study, all patients served as control groups and we collected only the standard preliminary data or none at all regarding patients going about their everyday lives as usual. Physical function, the need quality of life, discomfort, mental health, hospital stay length, and post-surgical status issues were taken into consideration as criteria for postoperative care [4]. One of the most common orthopedic treatments is THA. Future orthopedic surgeons must be trained in order to meet demand. Meanwhile, such training raises a number of challenging issues. There have been worries that procedures conducted by trainees may result in worse patient outcomes, a decline in efficiency, and an increase in medical delivery costs as a result [5].

Direct anterior THA

The direct anterior route to the hip for total arthroplasty has been said to have various advantages over other popular techniques (Table 1). Patients can adapt to the direct anterior approach (DAA) irrespective of body type and hip problems. It is understood that specific native hip and pelvic anatomical characteristics make a plain front process more challenging [6].

**Table 1:** Difference between THA and direct anterior THA THA, total hip arthroplasty

THA	Direct anterior THA
A THA is the type of surgery used to insert a full hip replacement	It is a method to fix a hip without causing tissue damage
It is the best invasive procedure for severe hip osteoarthritis	It can be done for all hip injuries
Advantage: reduces discomfort, improves movement, and brings back function	Advantage: less painful and fastest recovery from surgery
Disadvantage: leg length modification, loosening, nerve injury in the joint	Disadvantage: tingling in the thigh

Acetabular fracture in THA

THA is a widely used treatment for developed hip arthritis after acetabular fractures. A conservatively managed or operated condition may later lead to secondary arthritis necessitating a THA. Young individuals experience more acetabular fractures than their elderly counterparts. THA is acute or chronic in young or old patients with acetabular fractures. The surgeon must overcome some hurdles while performing a complete hip arthroplasty in an acetabular fracture, whether it is acute or delayed. The complications include fragments that prevent primary stability, amorphous anatomy, fibrosis and scar tissue between the pieces of bone, and potential bone fragment necrosis [7].

THA in femoral neck cracking

The ideal course of action for recent femoral neck fractures is still up for debate. The potential solutions include internal stabilization, HA, and total hip replacement (THA). THA is associated with better functional outcomes in independent, fit, and active patients, and a reduced chance of surgical repair. To prevent instability, a dual-mobility implant may be required [8]. Although there is a significant risk of instability following hip arthroplasty, whether complete (THA) or intermediate (HA), it is the standard for elderly patients with displaced intracapsular femoral neck fractures. Three different arthroplasties can be suggested: HAs and complete prosthesis without a dual mobility cup (DM THA) to reduce dislocation risk [9].

Spine rigidity in ankylosing spondylitis with THA in combined hips

The spine and hip joints are affected by ankylosing spondylitis (AS), which is characterized by progressive stiffness and function loss. Functional impairment is severe, involves the hip and spine, and is more common in younger age groups. Before THA, the spine's rigidity related to AS needs to be assessed. Hips with a THA fusion show flexion, abduction, or extension deformities. A flexion or extension deformity can be seen in the hip fused to the TTH. Because there is no typical pelvic movement pattern while moving from standing to sitting, people cannot sit properly. The lumbar spine, spinopelvic flexibility, and hip flexors are the three variables that influence how the spine moves from standing to sitting [10]. The prevalence and results of hip involvement in AS have been assessed in some sizable investigations. Hip involvement can be identified through clinical examination, radiographic analysis, MRI, or ultrasound [11].

Complications in THA

An ordinary total hip replacement problem is heterotopic ossification (HO). Not all patient groups experience the same level of prevalence. Hip ankylosis, male sex, and having experienced HO in the past are all considered risk factors at a considerable level. AP radiograph is used as the sole diagnostic tool. Male sex, fixed prostheses, double hip joint arthroplasty, ankylosing arthritis, and hip joint ankylosis are thought to enhance the incidence of non-articular ossification, according to a recent meta-analysis [12]. Nonsteroidal anti-inflammatory medications (NSAIDs) are frequently used for HO following THA prophylaxis. However, it is unclear if NSAIDs, especially selective NSAIDs as opposed to nonselective NSAIDs, are effective for treating HO. Many studies have shown the effectiveness of NSAIDs in preventing HO. NSAIDs are therefore frequently used for the prevention of HO. But surgeons have become concerned about the possibility of NSAIDs' gastrointestinal adverse effects [13].

THA and COVID-19 pandemic

COVID-19 is a highly contagious new coronavirus causing a worldwide epidemic. When COVID-19 symptoms manifest, patients frequently have lower respiratory symptoms such as fever, dry cough, weariness, muscle discomfort, and dyspnea. In addition to fractures, there are other possibly urgent surgical causes for THA that may call for early corrective surgery to reduce disability or the unnecessary harm that would be done to the patient if the operation was delayed [14]. Although the cancellation of elective surgery was necessary to conserve healthcare resources during the peak of the novel COVID-19 pandemic, recent data have shown that patients who had THA procedures cancelled are now experiencing worsening pain, a decline in physical activity, and an increase in anxiety [15]. To prevent the healthcare system from collapsing due to COVID-19, elective total joint arthroplasty procedures have been delayed. The majority of elective courses carried out in medical facilities around the world are THA treatments [16].

THA in the past, present, and future

Over the past three decades, there have been constant improvements in graft designs, biomedical techniques, surgical approaches, and knowledge of the physiological reconstruction of the hip, even though early designs had concerns with performance and even failure. These developments have improved THA clinical outcomes and implant survival rate.

Past

The author began his medical practice in 1990 with hybrid THA14, a low-friction form of THA3 that uses bonded and lacks a stable connection between the femoral and acetabular components. In the beginning, first-generation implants predominated. Several criteria were used to select the implants. An examination of the author's early series of 76 primary hybrid THAs with a mean take of 15.5 years found that there were 23 (30.3%) articular surface reoperations (separated acetabular liner transfer, 12 instances; cup revision, 11 cases) with an average lifetime to revision of 11.5 years (range: 14-19.5 years )[17]. In this inquiry, an inadequate cementing procedure mainly caused an early failure of the coated femoral stem. Good cement mantle achievement may increase the survival rates [18].

Present

Beginning in 2000, the author started using a second-generation acetabular cup to solve the difficulties brought up by the author in his prior experiences. Specifically, the second-generation acetabular cups have enhanced liner locking mechanisms and polished tapered femoral stems. In the author's study, 95 leading hybrid THAs with an average follow-up of 10 years were used. The excellent outcome of good implant survival and advance in a patient-reported result at the author's institute further show that THA is a workable substitute, especially for young individuals with symptomatic symptoms [17].

Future

Future THAs are anticipated to focus further on some areas. Current THAs have great mid- and long-term outcomes when bearings are made of alternative materials. Thus, we may now concentrate on functional result advancement after THAs, particularly in the people of Asia, a region where way of existence differs substantially from that of the western population [17].

Surgical challenges of THA in tubercular arthritis of the hip

Hip osteoarticular tuberculosis can be a crippling condition that leads to severe hip arthritis and extensive cartilage loss and destruction. THA is a potent cure option for individuals with severe post-tuberculous arthritis, but it has historically generated debate because of worries about the possibility of disease recurrence. THA offers pain alleviation as well as mobility and stability [19]. To cure coxotuberculosis, comparing the overall curative effects of total hip replacement and hip arthrodesis (HA) is essential. THA is a successful therapy for severe tuberculous arthritis. When treating coxotuberculosis, THA is superior to HA [20]. THA has a solid track record of efficacy as a therapy method for quiescent hip tuberculosis. THA is a safe treatment that offers clinical alleviation and functional outcomes in advanced active hip tuberculosis cases. Complete postoperative antituberculosis chemotherapy is the key to reducing the chance of tuberculosis recurrence [21].

Perioperative care in THA

Numerous heterogeneous research studies have addressed every aspect of total hip replacement surgery's improved surgical treatment. THA has a comparatively low rate of complications [22]. In total hip replacement, antibiotic prophylaxis should be standard practice; however, the selection of antibiotics should be based on price and local access [23]. The incidence of postoperative urine retention (POUR) after lower joint arthroplasty ranges from 0% to 75%. This wide range reflects the variations in POUR diagnosis and treatment [24]. When a multimodal analgesic regimen was delivered perioperatively in THA studies with intermediate risk, no extra analgesic impact of LIA relative to placebo was identified [25].

HIV in THA

THA should not be arbitrarily denied to HIV-positive patients. To avoid avoidable surgical problems, it is essential to identify all patients who are HIV positive and start them on HAART (highly active antiretroviral therapy) [26]. Life expectancy has risen as HIV medicines have advanced. As this group experiences pathological changes brought by ageing and avascular necrosis (AVN), the need for joint replacement is anticipated to rise (AVN) [27].

Trochanteric osteotomy in THA

The trochanteric osteotomy (TSO) technique allows for increased visibility and entry to the acetabulum and femoral canal for a range of factors. For both septic and disinfect revision, transfemoral approach , extended femoral trochanter osteotomy, and trochanteric slide osteotomy have been performed [28]. In femoral revised arthroplasty, the orthopedist frequently must choose between executing an expanded TSO and risking surgical fracture by trying to eliminate the femoral stem without an osteotomy [29]. A TSO has historically been necessary for successful glue removal and element reinsertion during THA [30].

THA for posttraumatic conditions

A significant portion of subsequent hip osteoarthritis is caused by posttraumatic arthritis [31]. The short-term efficacy of THA in treating posttraumatic osteoarthritis brought by acetabular rupture is excellent [32]. Uncemented THA can achieve good short-term effectiveness following transplant and is superior to cemented THA; yet, more analysis is required to regulate the efficacy over the medium and long term [33]. Uncemented THAs had a lower follow-up duration [34]. A wildly varying percentage of people who have received a kidney transplant have been documented to have femoral head osteonecrosis. Autoimmune reactions and poor bone quality increase the risk of aseptic release and infection when these patients undergo THA [35]. The treatment for AVN of the femoral head that is most frequently employed is total hip replacement [36].

## Conclusions

This review article described various surgical challenges or techniques that successfully helped treat THA according to their relations with other conditions. Many equally important factors are included in the review of the hip replacement procedure. Preoperative templating improves the precision of THA and other techniques. The management and understanding of surgical skills in THA are also essential. The trainee group's operational period was much more extended than the trainer groups. The trainee team's difficulty rate was more significant than the instructor teams. Other conditions such as tubercular arthritis, coxotuberculosis, COVID-19 pandemic, and acetabular fracture were studied in comparison to THA and collectively comprehended above. The past, present, and future of THA may be helpful for the everyday quality of life enhancement and physical function improvement.
